# Framing Effects and Evidence Type: Influence on the Persuasive Effect of Myopia Prevention Messages Among Elementary School Students in China

**DOI:** 10.3389/fpubh.2021.650879

**Published:** 2021-09-27

**Authors:** Zuyue Zhang, Yalan Lv, Qingmao Rao, Chunlv Ye, Ting Cheng, Mengyao Jiang, Li Bai, Xiaorong Hou

**Affiliations:** ^1^College of Medical Informatics, Chongqing Medical University, Chongqing, China; ^2^Medical Data Science Academy, Chongqing Medical University, Chongqing, China; ^3^Chongqing Engineering Research Center for Clinical Big-Data and Drug Evaluation, Chongqing, China; ^4^Nanping Sihai Elementary School, Chongqing, China; ^5^General Practice Ward, Children's Hospital of Chongqing Medical University, Chongqing, China

**Keywords:** myopia prevention, statistical evidence, non-statistical evidence, children, framing effects

## Abstract

**Background:** The myopia is a public health issue that attracts much attention. However, limited attention has been paid to the effect of primary school students' acceptance of health messages. Previous studies have found that framing effects and evidence types influence the persuasive effect of messages.

**Purpose:** This study explored whether framing effects and evidence type influence the persuasive effect of myopia prevention messages among elementary school students and the influence of children's myopia prevention cognition was considered.

**Methods:** A cross-sectional study was conducted among 1,493 elementary school students aged 9 to 13 in China from May to July 2020 by convenience sampling. Wilcoxon signed-rank test and multinomial logistic regression were used for data analysis.

**Results:** Significant differences were found in the persuasive effect between statistical and non-statistical evidence messages (*p* < 0.001). Among non-statistical evidence messages, gain-framed messages showed a greater persuasive effect than loss-framed messages (*p* < 0.001). Among statistical evidence messages, loss-framed messages performed better than gain-framed messages (*p* < 0.001). Children's myopia prevention cognition exerted no significant effect on the persuasive effect of the messages (*p* > 0.05).

**Conclusion:** This study demonstrated the influence of framing effect on the persuasive effect of myopia prevention messages among children aged 9 to 13 in China. Non-statistical evidence messages showed a better persuasive effect than statistical evidence messages. Different types of evidence influenced the persuasive effect of gain- and loss- framed messages. These findings have implications for strategies more or less likely to work in making myopia prevention messages for children.

## Introduction

Studies estimated that the global prevalence of myopia and high myopia will increase significantly by 2050, affecting nearly 5 billion people and 1 billion people, respectively (1). In recent years, the incidence of myopia among children and adolescents in China has been on the rise. According to the document The Healthy China Initiative 2019–2030, the overall myopia rate among children and adolescents was 53.6% in 2018; among them, children aged 6 years accounted for 14.5%, primary school students 36.0%, middle school students 71.6%, and high school students 81.0% (2). Vision is an important indicator of the physical health of children and adolescents, and myopia in elementary school students and adolescents in China is becoming more common at a younger age (3).

A large number of studies showed that the causes and influencing factors of myopia are complex and diverse, and genetic and environmental factors can affect the incidence of myopia., Lack of time for outdoor activities (4) and increased near work (5) are the risk factors that cause myopia, Overuse of electronic products is also likely to be a risk factor for myopia (6). Non-hereditary myopia can be prevented to a large extent by healthy and rational use of the eyes, but genetic susceptibility for myopia cannot be treated.

COVID-19 has spread worldwide since December 2019. In China, the isolation policy prevents students from returning to school, although some schools have begun organizing students to return to school when the epidemic was brought under control around April. Most students have spent a long period of online learning. During the COVID-19 outbreak, increased electronic use (mobile phones, tablets, TV sets, computers, and other tools) and reduced outdoor activities among children and adolescents increase the risk of myopia development and progression. In June 2020, the Ministry of Education of the People's Republic of China investigated the vision changes among primary, middle, and high school students in nine provinces (autonomous regions and municipalities directly under the Central Government) during the epidemic. Compared with the data at the end of 2019, the myopia rate of primary and middle school students increased by 11.7% in the 6 months (7).

Improving children's awareness of the healthy use of eyes to prevent myopia is important. Health information can help develop preventive behaviors (8). Information is an organic combination of substance and form. Under the same information content, the organizational form can be diversified. The effectiveness of information communication is closely related to the organizational form of information, and changing the organizational form of information can improve the acceptance of information and communication effect (9).

The framing effect is a reliable stimulus strategy first proposed by Tversky and Kahneman in 1981 (10) to improve the effectiveness of information transmission (11). Adopting appropriate frameworks in messages on specific topics can improve the acceptance of messages to the target population (12–15). On the basis of prospect theory, goal-framing effects suggest that messages have different powers of persuasion depending on how these messages are framed (16). A gain-framed message emphasizes the positive consequences of implementing an action, whereas a loss-framed message emphasizes the negative consequences of not implementing the intended action (17). Rothman argues that a loss-framed message is more acceptable than a gain-framed message in promoting detection behaviors, whereas a gain-framed message is more acceptable than a loss-framed message in promoting prevention behaviors (18). Gerend, Lithopoulos and Rosenblatt, respectively, found that a loss-framed message is more acceptable than a gain-framed message in terms of vaccination (prevention) behaviors, physical activity participation intention of patients with multiple sclerosis and healthy diet choice (prevention) behavior (9, 19, 20). The framing effect is an effective means to improve information inefficiency. Improving the involvement of the participants and using an appropriate framework to process the information of some topics can effectively improve the audience's acceptance of the information (11), and the health framing effect plays an important role in promoting healthy behaviors (21).

The type of evidence also affects the persuasive effect of the message (22, 23). Narrative and statistical evidence are more convincing than no evidence at all (24), and statistical evidence has a strong influence on beliefs and attitudes (25). A study with a sample size of 300 (Women with an average age of 24) in China has shown that differences in evidence may affect individuals' willingness to prevent cervical cancer (8). In the present study, we consider exploring the influence of goal-framing effects and evidence type on the persuasive effect of myopia prevention messages.

## Materials and Methods

### Study Design

A cross-sectional study about the persuasive effects of different framing messages of myopia prevention on children aged 9–13 years was conducted in China from May to July 2020. A self-administered paper questionnaire and an online questionnaire were used through a convenient sampling method. In the present study we have examined the persuasive effect of myopia prevention messages on children aged 9–13 years and the relationship between framing effects and evidence type. Basing on Prospect Theory, we compared the acceptance of children of the four types of simple myopia prevention messages with two result frames (loss- and gain-framed) × two types of evidence (non-statistical and statistical). We also investigated whether or not children's myopia prevention cognition affects the persuasive effect of the message.

### Participant Selection

The following are the inclusion criteria. The participants must be primary school students aged 9–13 years old because of their ability to read health information and it is an important period to prevent myopia before the age of 14 (26).

From several primary schools in Chongqing and Guangxi, China, a total of 1,493 participants were recruited using online and offline surveys, and the effective rate was 61.5%, the final valid questionnaires were 918.

### Questionnaire

The questionnaire was developed in accordance with existing literature and technical services specification for vision health management of primary and middle school students (T/CHAA 008-2019) (27), and the questionnaire was pilot tested before the final survey. The questionnaire was approved by a panel of experts. The questionnaire is divided into three parts: essential information (including demographic characteristics), children's myopia prevention cognition scale and myopia prevention message framing materials.

Demographic characteristics included the child's gender, age and grade. Information about the regular visual examination or not, average daily screen time, have eye exercises or not, myopia status and myopia of parents were collected.

The children's myopia prevention cognition was assessed by five questions: “Do you know that myopia will have a bad effect on your study and life? (1 = I don't know, 2 = I don't know very well, and 3 = I know very well)”; “What you think is the best reading distance for your eyes from a book? (1 = 30 cm, 0 = 25 cm, and 0 = 20 cm)” (28); “How long do you think using electronic products every day will harm our eyesight? (1 = 1 h, 0 = 30 min, and 0 = 2 h)” (2); “How much time do you think should you spend outdoors at least every day? (1 = 2 h, 0 = 1 h, 0 = 1 h 30 min)” (27, 29); “Can you use your knowledge of eye health to protect your eyes? (1 = I can't do it, 2 = I'm not sure, 3 = I can do it).” The scores of myopia prevention cognition among participants ranged from 2 to 9.

The content of framed messages in this study was designed based on previous studies, and relevant service specifications were referred to (27). Firstly, we identified two myopia prevention themes: average daily screen time and posture of reading and writing, and there are two messages for each different type of message. Then, as shown in Table 1, we designed four sets of framed messages in each theme: gain-framed and non-statistical evidence messages, gain-framed, and statistical evidence messages, loss-framed and non-statistical messages and loss-framed and statistical evidence messages. We used the mean score of same-type messages from different themes to represent the persuasive effect of a certain type of message.

**Table 1:** Message framing materials used in the study.

	**Gain- framed**	**Loss-framed**
Non-statistical evidence	(1) If we can control the use time of electronic products every day, then our eyesight will be protected, and the possibility of wearing short-sighted glasses will be reduced. (2) If we maintain the correct reading posture, we are less likely to be short sighted.	(1) If we use electronic products for a long time every day, we will likely suffer from myopia and may not be able to read words on the blackboard. (2) If we read with the wrong posture, our eyes will feel very tired after a long time, which will increase the possibility of myopia.
Statistical Evidence	(1) If we can control the total time of using electronic products within 1 h every day and the single-use time within 15 min, we can protect our eyesight. (2) If we keep our eyes more than 33 cm away from the book while reading, the possibility of myopia will be reduced by nearly 20%.	(1) If we use electronic products for more than an hour a day, we are more likely to develop myopia. (2) If we read with an incorrect posture, with our eyes <30 cm away from the book, or lying down, we are 74% more likely to be short-sighted after a long time.

### Data Collection

Demographic questions were answered by the participants. In the myopia prevention message framing materials, participants ranked four pieces of messages under each theme, with the first representing the most agreeable, the second representing the second most agreeable, the third representing the third most agreeable and the fourth representing the least agreeable. We assign the score for a persuasive effect to the ranking results (First Rank = Score 4, Second Rank = Score 3, Third Rank = Score 2, and Fourth Rank = Score 1). Therefore, the higher the score is, the better the persuasive effect is. The online questionnaire data were automatically recorded by the system, and the paper questionnaire data were input into the computer by the interviewers.

### Ethical Aspects

The study protocol was approved by the Ethics Committee of Chongqing Medical University (The record number is 2018011). Participants provided informed consent before completing the questionnaire.

### Statistical Analysis

Data were processed using Excel software before entry into the database. Data analysis was performed using SPSS 25.0 software (IBM Corporation, Armonk, NY, US). In the demographic characteristics of children, frequencies and percentages were calculated for categorical variables. We used the mean score to represent the persuasive effect of a certain type of message. Wilcoxon signed-rank test was performed to assess whether or not a significant difference exists between different types of messages. Multinomial logistic regression analysis was performed to investigate the factors that affect the persuasive effect of myopia prevention messages. Independent variables were evidence type, frame type, demographic characteristics, have a regular visual examination or not, average daily screen time, have eye exercises or not, myopia status, the myopia of parents and myopia prevention cognition. We have divided the messages into four groups (group 1, group 2, group 3, and group 4) according to the persuasive effect of the messages. The higher the number, the better the persuasive effect and group 1 served as the control group. A *p*-value not more than 0.01 was considered statistically significant.

### Quality Control

The questionnaire was modified several times after expert interviews and a pilot survey. The investigators received standardized training, had a detailed understanding of the purpose and methodology of the study and had extensive experience in dealing with potentially sensitive issues. In online surveys, the quality of questionnaire responses can be controlled by setting rules (for example, setting questions to the single choice to prevent subjects from selecting multiple answers or missing any questions).

## Results

### Demographic Characteristics of Participants

The demographic characteristics of the participants are presented in Table 2. There had 27.1% of the participants had myopia. The number of boys and girls in this study was basically the same (52.1% for boys and 47.9% for girls). Participants aged 12 years were the most (28.8%), and participants aged 9 years were the least (8%). Most of the participants were in the fourth and fifth grades (30.2 and 30.1%). More than half (62.5%) of the participants did not have a regular visual examination. In addition, 20.4% of the participants used electronic devices for more than 1 h per day. The mean daily sleep duration of the participants was 9.67 h (2.23 SD). The average children's myopia prevention cognition score (Maximum is 9) of the participants was 6.28 (1.42 SD).

**Table 2:** Demographic characteristics of children (*n* = 918).

**Variables**		**Sample percentage (*n*) or median (quartiles)**
Gender	Female	52.1% (440)
	Male	47.9% (478)
Age	9	8% (73)
	10	26.8% (246)
	11	18.6% (171)
	12	28.8% (264)
	13	17.9% (164)
Grade	Third	16.3% (150)
	Fourth	30.2% (277)
	Fifth	30.1% (276)
	Sixth	23.4% (215)
visual examination	Regular visual examination	37.5% (344)
	No regular visual examination	62.5% (574)
Average daily screen time	Not used	6.9% (63)
	Less than 15 min (including 15 min)	19.7% (181)
	15 minutes to half an hour (including half an hour)	27.1% (249)
	Half an hour to an hour (including an hour)	25.9% (238)
	Over an hour	20.4% (187)
Eye exercises	Do eye exercises regularly	47.6% (437)
	Not doing eye exercises on time	52.4% (481)
Myopia status	Myopia	27.1% (249)
	No myopia	72.9 (669)
Myopia of parents	Only father has myopia	10.8% (99)
	Only mother has myopia	13.1% (120)
	Both parents have myopia	8.2% (75)
	Neither parent has myopia	58.3% (535)
	Unknown	9.7% (89)
Myopia prevention cognition (Maximum is 9)		6 (5, 7)

### Persuasive Effect of Framing Messages

Two features of the messages were experimentally manipulated: goal framing and non-statistical vs. statistical evidence type. As shown in Table 3, we used the Wilcoxon signed-rank test to analyze the differences in the persuasive effect of different types of framing messages. A significant difference was observed between messages with non-statistical and statistical evidence (*p* < 0.001), and the persuasive effect of a message with non-statistical evidence was higher than that of a message with statistical evidence. However, no significant difference in persuasive effect was found between gain- and loss-framed messages (*p* = 0.517).

**Table 3:** Wilcoxon signed-rank test for testing persuasive effects (*n* = 918).

	**Median (Quartiles)**	***Z***	***P***
Gain	2.5 (2.25, 2.75)	−0.647	0.517
Loss	2.5 (2.25, 2.75)		
Non-statistical	2.75 (2.5, 3)	−13.410	0.000^**^
Statistical	2.25 (2, 2.5)		

*Wilcoxon signed-rank test was used,*

** *p < 0.001*.

### Moderating Effects Between Evidence Types and Framing Effects

Furthermore, we analyzed the moderating effects between evidence types and framing effects. As shown in Table 4, with different evidence types, the persuasive effects of gain- and loss-framed messages were significantly different (*p* < 0.001, *p* < 0.001). However, differences were found between messages with non-statistical and statistical evidence. Among the non-statistical evidence messages, the gain-framed messages showed a better persuasive effect than the loss-framed messages. However, among the statistical evidence messages, the opposite was true. Among the gain-framed messages, the non-statistical evidence messages showed a better persuasive effect than the statistical evidence messages.

**Table 4:** Persuasive effects of framed messages with different types of evidence.

		**Goal framing**		***Z***	***P***
		**Gain**	**Loss**		
Evidence type	Statistical	2 (1.5, 2.5)	2.5 (2, 3)	−7.612	0.000^**^
	Non-statistical	3 (2.5, 3.5)	2.5 (2, 3)	−7.571	0.000**
*Z*		−15.225	−2.853		
*P*		0.000 **	0.004		

*Wilcoxon signed-rank sum test was used,*

** *p < 0.001*.

### Multinomial Logistic Regression Analysis for the Factors Affecting the Persuasive Effects of Framed Message

Except for evidence type and framing type, no other independent variables in this study had a significant effect on the persuasive effect of myopia prevention messages. In particular, evidence type had a great impact on the persuasive effect of myopia prevention messages. As shown in Table 5, Some of the factors are shown (group 1 served as the control group), similar to our previous analysis, the non-statistical evidence messages showed better persuasive than the statistical evidence messages, group 2 (OR, 95%CI: 1.73, 1.51–1.97), group 3 (OR, 95%CI: 3.31, 2.89–3.79), group 4 (OR, 95%CI: 2.57, 2.25–2.94). In addition, children's myopia prevention cognition had no significant effect on the persuasive effect of the message, group 2 (OR, 95%CI: 1.00, 0.95–1.05), group 3 (OR, 95%CI: 1.00, 0.95–1.05), group 4 (OR, 95%CI: 1.00, 0.95–1.05).

**Table 5:** Multinomial logistic regression analysis for factors affecting persuasive effect.

**Score^a^**			***B***	***P***	**OR**	**95%CI**	
2	Cognition		0.000	0.991	1.00	0.95	1.05
	Age		−0.001	0.970	1.00	0.95	1.06
	Gender	Male	0.003	0.969	1.00	0.88	1.14
		Female	0^b^	–	1.00	–	–
	Average daily screen time	Not used	−0.001	0.996	1.00	0.75	1.34
		Less than 15 min (including 15 min)	0.000	0.998	1.00	0.81	1.24
		15 min to half an hour (including half an hour)	0.006	0.949	1.01	0.83	1.22
		Half an hour to an hour (including an hour)	−0.001	0.988	1.00	0.82	1.21
		Over an hour	0^b^	–	1.00	–	–
	Myopia status	Myopia	0.005	0.950	1.01	0.86	1.17
		No myopia	0^b^	–	1.00	–	–
	Myopia of parents	Only father has myopia	−0.008	0.946	0.99	0.80	1.24
		Only mother has myopia	0.002	0.985	1.00	0.82	1.23
		Both parents have myopia	−0.004	0.975	1.00	0.77	1.28
		Unknown	0.005	0.968	1.01	0.80	1.26
		Neither parent has myopia	0^b^	–	1.00	–	–
	Goal framing	Gain	−0.082	0.214	0.92	0.81	1.05
		Loss	0^b^	–	1.00	–	–
	Evidence type	Non-statistical	0.547	0.000	1.73	1.51	1.97
		statistical	0^b^	–	1.00	–	–
3	cognition		0.000	0.999	1.00	0.95	1.05
	Age		−0.001	0.966	1.00	0.94	1.06
	Gender	Male	0.000	0.997	1.00	0.88	1.14
		Female	0^b^	–	1.00	–	–
	Average daily screen time	Not used	−0.001	0.993	1.00	0.74	1.34
		Less than 15 min (including 15 min)	0.000	0.998	1.00	0.81	1.24
		15 min to half an hour (including half an hour)	0.006	0.954	1.01	0.83	1.23
		Half an hour to an hour (including an hour)	0.002	0.980	1.00	0.82	1.22
		Over an hour	0^b^	–	1.00	–	–
	Myopia status	Myopia	0.006	0.941	1.01	0.86	1.17
		No myopia	0^b^	.	1.00	.	.
	Myopia of parents	Only father has myopia	−0.003	0.979	1.00	0.80	1.25
		Only mother has myopia	0.006	0.956	1.01	0.82	1.24
		Both parents have myopia	−0.005	0.973	1.00	0.77	1.29
		Unknown	0.005	0.967	1.01	0.80	1.27
		Neither parent has myopia	0^b^	–	1.00	–	–
	Goal framing	Gain	−0.408	0.000	0.66	0.58	0.76
		Loss	0^b^	–	1.00	–	–
	Evidence type	Non-statistical	1.196	0.000	3.31	2.89	3.79
		statistical	0^b^	–	1.00	–	–
4	cognition		0.000	0.987	1.00	0.95	1.05
	Age		−0.001	0.968	1.00	0.94	1.06
	Gender	Male	0.002	0.972	1.00	0.88	1.14
		Female	0^b^	–	1.00	–	–
	Average daily screen time	Not used	−0.001	0.995	1.00	0.74	1.34
		Less than 15 min (including 15 min)	0.002	0.998	1.00	0.81	1.24
		15 min to half an hour (including half an hour)	0.004	0.965	1.00	0.83	1.22
		Half an hour to an hour (including an hour)	0.009	0.926	1.01	0.83	1.23
		Over an hour	0^b^	–	1.00	–	–
	Myopia status	Myopia	0.005	0.953	1.01	0.86	1.17
		No myopia	0^b^	–	1.00	–	–
	Myopia of parents	Only father has myopia	0.002	0.989	1.00	0.80	1.25
		Only mother has myopia	0.001	0.995	1.00	0.82	1.23
		Both parents have myopia	−0.004	0.975	1.00	0.77	1.28
		Unknown	0.010	0.934	1.01	0.80	1.27
		Neither parent has myopia	0^b^	–	1.00	–	–
	Goal framing	Gain	0.053	0.433	1.05	0.92	1.20
		Loss	0^b^	–	1.00	–	–
	Evidence type	Non-statistical	0.943	0.000	2.57	2.25	2.94
		statistical	0^b^	–	1.00	–	–

a *The reference category is: 1*.

b *This parameter is set to zero because it is redundant*.

## Discussion

In this study, we aimed to explore how to improve the persuasive effects of myopia prevention messages on children aged 9–13 years. No significant difference was found between the persuasive effect of gain-framed and loss-framed messages, but a significant difference was found between messages with non-statistical and statistical evidence, for example, when we describe that “the probability of suffering from myopia will greatly increase,” the persuasive effect of messages is better than “the probability of suffering from myopia will increase by 70%”. The design of the questionnaire referred to previous studies (11, 12) and relevant vision health management documents issued in China (13, 14), and referred to previous studies on framed messages (15, 16) when designing message framing materials.

Different from some previous studies, in the present study we found that the framing effect did not affect the persuasive effect of the messages. Previous studies found that gain-framed messages might be more persuasive than loss-framed messages for some prevention behaviors and loss-framed messages might be more persuasive than gain-framed messages for some health detection behaviors (30–33). However, the meta-analysis also showed that no statistically significant differences in persuasiveness between gain- and loss-framed messages in some preventive actions (such as safer-sex behaviors, skin cancer prevention behaviors, or diet and nutrition behaviors) and vaccination action (34, 35), the results of the present study confirm these views to a certain extent Unlike previous studies, we have also innovatively added evidence type as an independent variable influencing the persuasive effect of messages. In the later part of the study, we explored the influence of evidence type on message framing. This study can provide some references for future research on the framing effect, and future researchers can consider studying the framing effect together with other factors that influence the persuasive effect of messages.

Non-statistical evidence messages showed a better persuasive effect in our study. Empirical studies found that statistical evidence can improve the persuasiveness of messages and that statistical evidence messages can enhance systematic and heuristic processing (24, 36, 37). However, in the present study, we obtained a different result. We found that the type of evidence was related to the persuasive effect of the myopia prevention messages on primary school students, but the non-statistical evidence messages showed a better persuasive effect than the statistical evidence messages. Because most previous studies did not select children but older subjects, we suspect that the reason for this result may be that children may be less sensitive to statistical evidence, they are more likely to accept non-statistical evidence because of their limited cognitive level (38, 39).

Furthermore, a moderating effect was found between evidence type and the framing effect. To further investigate the relevance of the framing effect and evidence type, we divided the messages into two groups according to evidence type and then investigated the difference in persuasive effect between gain-and loss-framed messages. We have found that in the messages containing different types of evidence, the influence of goal framing will change. This difference may be due to a change in evidence type. Different from some previous studies (40), this study proves that evidence type has a moderating effect on the framing effect possible because of the different research groups.

We also investigated whether or not children's myopia prevention cognition influences the persuasive effect of messages. As shown in Table 5, children's myopia prevention cognition exerted no significant influence on the persuasive effects of four types of messages. This result is unexpected, which may be related to the young age of the participants.

This study has certain limitations. Firstly, we used a convenient sampling method in the experimental design to ensure that the participants in the study are willing to read our information materials. However, we increased the contingency and reduced the representativeness of the sample. Secondly, the participants were between 9 and 13 years old, and they were not representative of children of all ages. Thirdly, other factors that may affect vision (beyond screen exposure time and near work) were not included in the study because the length of the questionnaire was limited, and parents' education level may be a confounder variable that was not considered in the study. Finally, the study relied on self-report, which can introduce bias because of dishonesty, over-reporting, under-reporting and measurement flaws. Limitations of this study would provide interesting avenues for further research. Despite these limitations, this study provided insights into the development of myopia prevention messages for children aged 9–13 in China.

## Conclusion

Messages with non-statistical evidence had a better persuasive effect than messages with statistical evidence. Further analysis revealed that evidence type exerted a moderating effect on the framing effect. The gain-framed messages showed a better persuasive effect than the loss-framed messages among the messages with non-statistical evidence, whereas the loss-framed messages had a better persuasive effect than the gain-framed messages among the messages with statistical evidence. Children's myopia prevention cognition exerted no significant impact on the persuasive effect of messages. The results of this study will contribute to the formulation of children's myopia prevention messages and improve the persuasive effect of children's myopia prevention messages.

## Data Availability Statement

The raw data supporting the conclusions of this article will be made available by the authors, without undue reservation.

## Ethics Statement

The studies involving human participants were reviewed and approved by Ethics Committee of Chongqing Medical University. Written informed consent to participate in this study was provided by the participants' legal guardian/next of kin.

## Author Contributions

XH, ZZ, QR, TC, and LB designed the experiments. XH, ZZ, QR, CY, and MJ performed the experiments. ZZ wrote the paper and analyzed the data. YL helped analyse the data. XH and YL helped draft the manuscript. All authors have read and approved the final manuscript.

## Funding

This research was funded by the Ministry of Education in China Project of Humanities and Social Sciences (Grant No. 20YJCZH043); the Intelligent Medicine Research Project of Chongqing Medical University in 2019 (Grant No. ZHYX2019006); Chongqing Medical University, China, the Intelligent Medical Foundation of Chongqing Medical University (Grant No. YJSZHYX202016); the College of Medical Informatics, Chongqing Medical University, China, Student Research and Innovation Experiment Project (Grant No. 2019C009); Chongqing Municipal Education Commission, China, Chongqing Graduate Scientific Research Innovation Project (Grant No. CYS20225).

## Conflict of Interest

The authors declare that the research was conducted in the absence of any commercial or financial relationships that could be construed as a potential conflict of interest.

## Publisher's Note

All claims expressed in this article are solely those of the authors and do not necessarily represent those of their affiliated organizations, or those of the publisher, the editors and the reviewers. Any product that may be evaluated in this article, or claim that may be made by its manufacturer, is not guaranteed or endorsed by the publisher.
